# Limited referral to nephrologists from a tertiary geriatric outpatient clinic despite a high prevalence of chronic kidney disease and anaemia

**DOI:** 10.1186/1471-2318-12-43

**Published:** 2012-08-03

**Authors:** Neil Boudville, Kalindu Muthucumarana, Charles Inderjeeth

**Affiliations:** 1 School of Medicine and Pharmacology, University of Western Australia, Verdun Street, Nedlands, WA, Australia; 2 Department of Renal Medicine, Sir Charles Gairdner Hospital, Verdun Street, Nedlands, WA, Australia; 3 Department of Aged Care and Rehabilitation, Sir Charles Gairdner Hospital, Verdun Street, Nedlands, Western Australia

**Keywords:** Anaemia, Chronic kidney failure, Geriatrics, Referral and consultation

## Abstract

**Background:**

Chronic kidney disease (CKD) is increasing in prevalence world-wide with the largest growth being in the elderly. The aim of this study was to examine the prevalence of CKD in a geriatric outpatient clinic within a tertiary hospital and its association with anaemia and mortality with a focus on the referral patterns towards nephrologists.

**Methods:**

Retrospective study utilising administrative databases. The cohort was defined as all patients that attended the geriatric outpatient clinics of a single tertiary hospital within the first 3 months of 2006. Patients were followed for 18 months for mortality and referral to a nephrologist.

**Results:**

The mean Glomerular filtration rate (eGFR) of the 439 patients was 67.4 ± 29.1 mL/min/1.73 m^2^ (44% <60 mL/min/1.73 m^2^). 11.8% had a haemoglobin < 110 g/L, with anaemia being significantly associated with kidney function in those with a eGFR < 60 mL/min/1.73 m^2^ (p = 0.0092). Kidney function and anaemia were significantly associated with mortality on multivariate analysis (p = 0.019 and p = 0.0074). After 18 months, 8.8% of patients with CKD were referred to a nephrologist.

**Conclusion:**

Despite a high prevalence of CKD in patients attending a geriatric outpatient clinic and its association with anaemia and mortality, few of these patients were referred to a nephrologist. An examination of the reasons behind this bias is required.

## Background

Chronic kidney disease (CKD) is a growing problem due to our ageing population, many of who have increased comorbidities [1]. Cross-sectional studies in the general population demonstrate that CKD is seen in up to 15.3%, including 39% of those over the age of 65 years [1-3]. Previously restrictive selection criteria for commencement of dialysis in a patient have over the years been relaxed, for a number of reasons. As a consequence prevalent dialysis numbers are increasing with its associated considerable cost [1,4].

In addition to the cost of dialysis, earlier stages of CKD are associated with increased morbidity and mortality [5,6]. A common complication, even with mild to moderate CKD, is anaemia [7,8]. This has significant effects on the quality of life of an individual that can be improved through the use of epoetins [9].

There is a perception that medical professionals caring for the elderly are potentially biased against referring patients to Nephrologists [10,11]. This may be partly due to the strict selection criteria that existed, when renal replacement therapy initially began, for the commencement of dialysis which often included an age limit[11]. In addition, some health care professionals may view that quality of life for their elderly patients may be better served by not going onto dialysis.

Our aim was to examine the prevalence of CKD in a tertiary Geriatrics outpatient clinic, in addition to its association with anaemia and mortality, and the referral patterns of Geriatricians to Nephrologists.

## Methods

This was a retrospective study that included all patients over the age of 65 years who were reviewed in the outpatient clinics of the Department of Aged Care and Rehabilitation in Sir Charles Gairdner Hospital (SCGH) during the first quarter of 2006. This is the second largest teaching hospital within Western Australia with clinics including general geriatrics clinics, and specialised memory and falls clinics. The Department of Aged Care and Rehabilitation included 6 Specialists. There were 5 Specialists in the Department of Renal Medicine in SCGH with 20 overall in Western Australia. Patients with end-stage kidney disease were excluded. This study was approved by the hospital Human Research and Ethics Committee.

The hospital laboratory database (Pathwest) was then examined for the results of blood tests performed on these patients. In addition, medical records were reviewed. Variables recorded included demographic data, serum creatinine, haemoglobin, and iron studies. Referral to a nephrologist within the next 18 months was then recorded through review of the patients’ medical records, CKD databases, and review of individual nephrologist databases. Survival after 18 months was also recorded.

Creatinine was measured using a kinetic colourimetric assay (Jaffe method) analysed on the Roche Hitachi 917 analyser (Roche Diagnostic GmbH, Mannheim, Germany). Total CV’s for this assay are 6.6% at 0.8 mg/dL, and 4.1% at 5.5 mg/dL. Glomerular filtration rate (eGFR) was estimated from serum creatinine utilising the 4-variable Modification of Diet in Renal Disease study equation [12].

All variables are reported as mean ± standard deviation, unless otherwise stated. Normality of variables was examined and if required transformation to improve normality was performed. Patients were grouped based upon their age into one of 3 categories – 65–75 years old; 75–85 years old; and over 85 years old. Simple linear regression was utilised to assess the relationship between haemoglobin and eGFR. Cox proportional hazards regression were used to examine the association between mortality and kidney function and haemoglobin on univariate and multivariate analysis. Other variables were examined to detect an association with mortality including age, gender, and iron status. Variables with a p-value < 0.10 on univariate analysis was added in the multivariate model. STATA 11.0 (Stata Corporation, College Station, Texas, USA) was used for the analysis.

## Results

439 patients were reviewed during the first 3 months of 2006 and had biochemical testing performed (Table 1). A total of 867 patients were reviewed in this clinic during the baseline period. The mean age of the total 867 patients was 80.2 ± 8.5 years, compared to 80.7 ± 8.9 years for those with biochemical data. 38.6% of the total population was male compared with 39.9% in those with a serum creatinine measurement.

**Table 1:** Baseline demographics

	**65-75 years old (n = 87)**	**75-85 years old (n = 221)**	**>85 years old (n = 131)**	**All (n = 439)**
Male (%)	42 (47.1%)	89 (40.3%)	44 (33.6%)	175 (39.9%)
Estimated Glomerular Filtration Rate (eGFR) (mL/min/1.73 m^2^)	74.0 ± 26.7	69.4 ± 31.8	59.7 ±24.2	67.4 ± 29.1
Chronic Kidney Disease (CKD) stage (eGFR) (%)				
1 (>90 mL/min/1.73 m^2^)	33.3	17.2	11.5	18.7
2 (60–90 mL/min/1.73 m^2^)	34.5	44.8	26.7	37.4
3 (30–60 mL/min/1.73 m^2^)	26.4	33.9	56.5	39.2
4 (15–30 mL/min/1.73 m^2^)	2.3	3.2	5.3	3.6
5 (<15 mL/min/1.73 m^2^)	3.5	0.9	0	1.1
Haemoglobin (g/dL)	13.0 ± 1.7	13.0 ± 1.8	12.6 ± 1.9	12.9 ± 1.8
Mean Cell Volume (fL)	91.3 ± 4.9	91.5 ± 6.7	92.8 ± 6.1	91.9 ± 6.2
Vitamin B12 (pmol/L)	410.9 ± 203.1	409.3 ± 320.0	436.8 ± 360.2	418.1 ± 318.3
Red cell folate (nmol/L)	1087.9 ± 653.8	1477.6 ± 940.9	1082.1 ± 871.3	1294.2 ± 898.3
Ferritin (μg/L)	251.9 ± 491.6	221.3 ± 342.9	169.3 ± 228.2	212.4 ± 345.9
Transferrin saturation (%)	21.6 ± 9.0	17.2 ± 10.2	16.9 ± 8.8	17.9 ± 9.7

### CKD

44% of patients had a eGFR < 60 mL/minute/1.73 m^2^, with 20%, 5% and 1.1% having a eGFR <45 mL/min/1.73 m^2^, <30 mL/min/1.73 m^2^ and <15 mL/min/1.73 m^2^ respectively. The mean eGFR for people aged 65–75 years was 74.0 ± 26.7 mL/min/1.73 m^2^, 75–85 years was 69.4 ± 31.8 mL/min/1.73 m^2^, and for those over 85 years was 59.7 ± 24.2 mL/min/1.73 m^2^.

### Anaemia

**Figure 1:**
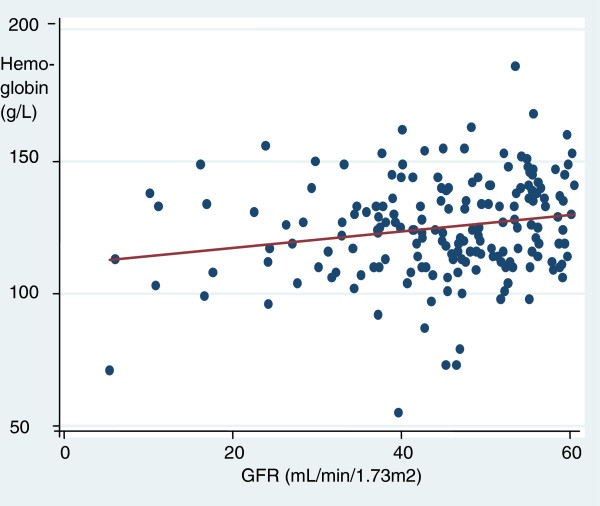
**Scatter plot and regression line for estimated Glomerular filtration rate (eGFR) versus haemoglobin in those participants with a eGFR < 60 mL/min/1.73 m**^**2**^.

11.8% of this sample had a haemoglobin less than 110 g/L, with 4.3% having a haemoglobin less than 100 g/L. Of those with a eGFR < 60 mL/min/1.73 m^2^, 17.2% had a haemoglobin <110 g/L and 6.5% had a haemoglobin <100 g/L. Haemoglobin was not significantly associated with eGFR on linear regression in the total population (p = 0.065), but it did reach significance on examining those with a eGFR < 60 mL/min/1.73 m^2^ (Figure 1, p = 0.0092). Mean haemoglobin was similar between those aged 65-75 years, 75-85 years and over 85 years at 130 ± 17 g/L, 130 ± 17 g/L and 126 ± 19 g/L respectively. The mean ferritin was 94 ± 154 ng/mL with a transferrin saturation of 17.9 ± 9.7%.

### Mortality

After 18 months of follow-up, 16.0% of the sample had died, compared to 10.3% of the total 867 patients that attended the clinics. Death was significantly associated with eGFR and anaemia on univariate analysis, p = 0.0074 and p = 0.019 respectively (Table 2). On multivariate logistic regression, both eGFR and haemoglobin remained independently associated with mortality, p = 0.0047. Survival analysis indicated that CKD stage 4 was associated with a 19.7 fold increased hazard of mortality than CKD stage 1, p = 0.012.

**Table 2:** Univariate and multivariate logistic regression of factors associated with death.

**Variable**	**Univariate analysis (p-value)**	**Multivariate analysis (p-value)**
Haemoglobin (g/L)	0.02	0.03
Glomerular filtration rate (mL/min/1.73 m2)	0.01	0.03
Age (years)	0.009	0.36
Gender	0.03	0.06

### Referral to Nephrologist

After 18 months of follow-up, 17 patients were referred to a nephrologist. This represents 8.8% of those patients with known/identified CKD on blood test. 34 patients had, at baseline, either a haemoglobin <100 g/L or a eGFR > 60 mL/min/1.73 m^2^. The mean eGFR of those patients referred to a nephrologist was 27.1 ± 14.2 mL/min/1.73 m^2^, with 10 (58.8%) having a eGFR < 60 mL/min/1.73 m^2^ (see Table 3). Haemoglobin, age and eGFR did not predict referral to a nephrologist, p = 0.93, 0.95 and 0.055 respectively.

**Table 3:** Age and Chronic Kidney Disease (CKD) stage of patients referred to a nephrologist

**CKD stage (eGFR)**	**Age = 65–75 years**	**Age = 75–85 years**	**Age > 85 years**
1 (>90 mL/min/1.73 m^2^)	0%	0 %	0%
2 (60–90 mL/min/1.73 m^2^)	0%	0%	0%
3 (30–60 mL/min/1.73 m^2^)	0%	29.4%	23.5%
4 (15–30 mL/min/1.73 m^2^)	5.9%	17.6%	5.9%
5 (<15 mL/min/1.73 m^2^)	11.8%	5.9%	0%

## Discussion

Chronic kidney disease is associated with increased morbidity, mortality, haematological and biochemical abnormalities. Increasing age is associated with an increased prevalence of CKD [3]. There is evidence however that there may be a pattern of restricted referral to Nephrologists from medical practitioners caring for the elderly [10,11]. Our study demonstrated that CKD is common in patients over 65 attending a tertiary geriatrics outpatient clinic. CKD was associated with anaemia and mortality but despite this few patients were referred to a nephrologist.

Mortality has been demonstrated to be inversely associated with kidney function, with the risk increased once the eGFR is below 60 mL/min/1.73 m^2^[5]. Interventions that can slow progressive renal failure exist but sometimes require input from a nephrologist [13,14]. In addition a nephrologist can assist with the optimal management of mineral bone disease that also occurs with increasing prevalence once eGFR < 60 mL/min/1.73 m^2^[15-17].

Anaemia can be directly related to CKD, with its increased risk commencing once the eGFR reaches 60 mL/min/1.73 m^2^ or less [7,8]. This was demonstrated in our study with an 11.3% prevalence of anaemia in our population. Quality of life can be improved considerably, and blood transfusions avoided, with the prescription of erythropoiesis stimulating agents [18,19]. In Australia, government subsidised prescription of these agents is restricted to Nephrologists and Haematologists.

The prevalence of CKD with a eGFR < 60 mL/min/1.73 m^2^ in our geriatric outpatient population was 44%, with an increased rate with increasing age. This is consistently greater than the prevalence of CKD in other cohorts - 14.7% of over 65 year old residents of long-term care facilities, 15.6% in outpatient clinics, 17.5% in a community-based laboratory, and 22.4% over 65 year olds in a primary care setting [5,20-22]. Other studies have shown that a considerable proportion of patients do not receive evidence based intervention for CKD in non-nephrology practices [23]. Suggesting that referral of this population to a Nephrologist may assist in the diagnosis of the aetiology of the CKD and to ensure that optimal management was being prescribed.

Contrary to the belief of some medical practitioners, commencement of dialysis in the elderly may be associated with an improved quantity and quality of life [24]. As a consequence of multiple factors, the greatest increase in new dialysis patients in Australia and the UK are in the over 65 age group [1,25]. Late referral to a nephrologist may delay optimal preparation for dialysis and is associated with worse outcomes, prompting national and international Guidelines for early referral of CKD patients to Nephrologists [24,26,27].

Despite this there was a surprisingly low referral rate of patients with CKD to a Nephrologist, 50% of the recommended numbers based upon Kidney Health Australia guidelines of eGFR < 30 mL/min/1.73 m^2^ or Haemoglobin < 100 g/L with eGFR = 30-60 mL/min/1.73 m^2^[26,28,29]. It is unclear why this practice pattern exists [28]. It may be related to a previous bias towards not dialysing the elderly or the belief that dialysis is not appropriate or in the best interests of the elderly.

The Nephrology workforce in SCGH and in the State is similar to the rest of Australia, with 72.7 end-stage kidney disease (ESKD) patients per full-time equivalent nephrologist in WA compared with 60.3 for Australia [30]. In addition the demographics of ESKD patients in WA is similar to the rest of Australia with 453 Dialysis patients per million population, compared with 471 in Australia [31]. The peak in the age group for prevalent dialysis patients in WA and Australia is also the same at 65 to 74 years old [31]. This suggests that results from this paper may be generalizable to the rest of Australia.

Limitations include its retrospective nature. While a complete list of people attending the geriatrics outpatients clinic during the baseline period was obtained, data was only obtained on those patients that were having blood tests performed for clinical reasons. This bias is likely to overestimate the true prevalence rates. Additionally, an examination was not made to see if the CKD patients identified were already on optimal treatment to slow progressive kidney failure. Regardless, those with anaemia and advanced CKD would probably benefit from referral to a nephrologist. Other metabolic complications of CKD were not examined due to the lack of information as measurement of these parameters (e.g. serum phosphate) were not part of usual clinical practice in these clinics.

## Conclusions

We have documented a high prevalence of CKD in patients attending a tertiary hospital geriatrics outpatient clinic. The CKD was associated with an increased risk of mortality and anaemia. The referral patterns of the supervising practitioners demonstrated restricted consultation with nephrologists, despite the potential benefits to the patient. This requires further investigation to examine the reasons behind these referral patterns in order to be able to implement interventions to increase referral if it is deemed appropriate.

## Competing interest

The authors declare that they have no competing interests.

## Authors’ contributions

NB was involved in the design of this study, analysis of the dataset and drafted this manuscript. KM assisted with the design of this study, collected the data from medical records and administrative databases and reviewed the manuscript. CI assisted with the design of this study and reviewed the manuscript. All authors read and approved the final manuscript.

## Funding

Self-funded by the Department of Renal Medicine, SCGH. No authors received any personal funds for their contribution to this manuscript.

## Pre-publication history

The pre-publication history for this paper can be accessed here:

http://www.biomedcentral.com/1471-2318/12/43/prepub
